# Depression and Self-Efficacy Among Iranian Children During the Prevalence of COVID-19 Disease

**DOI:** 10.3389/fped.2022.888712

**Published:** 2022-06-30

**Authors:** Mohammad Ali Zakeri, Abdollah Dakkalirad, Fahimeh Saedi, Allahyar Shahnavazi, Mehri Kordi, Maryam Ahmadipour, Mahlagha Dehghan

**Affiliations:** ^1^Social Determinants of Health Research Center, Rafsanjan University of Medical Sciences, Rafsanjan, Iran; ^2^Non-communicable Diseases Research Center, Rafsanjan University of Medical Sciences, Rafsanjan, Iran; ^3^Tropical and Communicable Disease Reasearch Center, Iranshahr University of Medical Sciences, Iranshahr, Iran; ^4^Shafa Hospital, Kerman University of Medical Sciences, Kerman, Iran; ^5^School of Nursing and Midwifery, Iranshahr University of Medical Sciences, Iranshahr, Iran; ^6^Nursing Office, Iranshahr University of Medical Sciences, Iranshahr, Iran; ^7^Department of Pediatric, Afzalipour Hospital, Afzalipour Faculty of Medicine, Kerman University of Medical Sciences, Kerman, Iran; ^8^Department of Critical Care Nursing, Razi Faculty of Nursing and Midwifery, Kerman University of Medical Sciences, Kerman, Iran

**Keywords:** COVID-19, coronavirus, depression, self-efficacy, children

## Abstract

The coronavirus disease 2019 (COVID-19) outbreak has quickly endangered the physical and mental health of people in the community, particularly vulnerable people such as children. This study was carried out to investigate the depression and self-efficacy of Iranian children during the COVID-19 outbreak. This cross-sectional research was conducted on 321 students aged 8 to 17 in southeast Iran. A social media-based online questionnaire was used to collect data. The information was gathered using demographic and COVID-related items, the Children's Depression Inventory (CDI), and the Self-Efficacy Questionnaire for Children (SEQ-C). No significant correlation was observed between depression and self-efficacy of children (*P* = 0.23). However, in subscale of CDI, negative mood, ineffectiveness and negative self-esteem had a significant correlation with self-efficacy (<0.001). Depression had a significant correlation with family income (*p* = 0.017), being at risk of coronavirus infection (*p* = 0.036), effectiveness of preventive measures (*p* = 0.015) and how information about the coronavirus disease was obtained (*p* = 0.018). According to the results, the mean score of depression was higher than the midpoint of the questionnaire in Iranian children, therefore, it is needed to take the necessary measures and treatment plans to reduce the rate of depression in children. Further research is needed to assess and prevent childhood depression.

## Introduction

Coronavirus is a type of virus that can cause respiratory infections. The virus can cause mild illnesses like colds or more severe illnesses like the severe acute respiratory syndrome (SARS) and the Middle East respiratory syndrome (MERS). The virus is now known as COVID-19 (1). The World Health Organization (WHO) has classified the disease as a major threat to physical and mental health because the outbreak of COVID-19 disease has altered families' daily and normal lives, particularly their lifestyle (2). COVID-19 has also spread in Iran, threatening people's physical and mental health (3), as the disease prevalence has raised concerns about the possibility of death from viral infections and has caused psychological stress (4).

On the other hand, the spread of COVID-19 resulted in home lockdown and the closure of many recreational and educational facilities, which had a negative impact on people's mental health (5). School closures and home quarantine of students due to the spread of COVID-19 disease have a negative impact on children's physical and mental health (6). Reduced physical activity in children, the emergence of stressful stimuli at home, such as fear of getting sick, unpleasant thoughts, lack of communication with peers and friends, and lack of space for physical activity at home, as well as parental fear and anxiety, can all have long-term effects on children's mental health (7).

Children have to stay at home for a long period due to mandatory isolation and school closures during the COVID-19 outbreak, resulting in limited contact with peers, reduced physical activity, as well as more behavioral and emotional problems among them (8). Furthermore, home quarantine and its association with other factors such as parental mental illness and substance abuse, low socioeconomic status of the family, exposure to domestic violence, and a lack of opportunities for play and entertainment can have a significant impact on children's mental health during the COVID-19 epidemic (9). Children who witness the disease in others, on the other hand, are more likely to develop disorders like posttraumatic stress disorder and insomnia (10).

The limitations of COVID-19 disease can lead to adverse physical and mental health consequences. Children are among the most vulnerable groups who are at greater risk in such stressful situations.

In general, the prevalence of COVID-19 can cause feelings of insecurity, anxiety, fear, depression, insomnia, behavioral problems, irritability, posttraumatic stress disorder, and obsessive-compulsive disorder in children (11). Recent coronavirus research has found that anxiety caused by the coronavirus and family quarantine endanger family mental health and make children more susceptible to symptoms of psychological disorders (6, 12). Ghosh et al. (11) discovered that children were more likely to experience psychological problems during a crisis, particularly an epidemic. According to Wang et al. (13), children suffer more psychologically from an epidemic and experience a psychological crisis, which leads to a variety of psychological problems such as anxiety and depression. According to research, higher self-efficacy is associated with better practice (14) and lower psychological distress (15). The results of Zhou et al. (16) on the prevalence of coronavirus showed that people with higher general self-efficacy were more likely to have lower risk perceptions, fewer passive coping strategies, more active coping strategies, and subsequently fewer mental health problems. According to the results obtained from some research, fear of COVID-19 increases communication anxiety and decreases self-efficacy (17).

Self-efficacy is a person's belief in his or her ability to perform a specific task. This concept overshadows the person's effort and practice and refers to one's judgement of one's ability to perform an action, which can enable a person to adopt health-promoting behaviors and discontinue health-harming behaviors (18). Albert Bandura's cognitive-social theory is based on the concept of self-efficacy, which refers to people's beliefs in their capabilities to exercise control over their own functioning and over events that affect their lives. Bandura (19) believed that one of the most important factors in regulating human behavior was self-efficacy. Self-efficacy refers to an individual's belief in his or her ability and capacity to execute behaviors necessary to produce specific performance attainments. It means the belief a person has in their ability to attain results, to meet the challenges ahead of them, and to influence events that effect their own lives. Self-efficacy is predictive of future behavior, and it can serve as the inducer or inhibitor of appropriate actions (20). Hong et al. (21) acknowledged that the emotional climate of the classroom could have a significant impact on social self-efficacy, that social self-efficacy could significantly predict self-esteem and depression, and that self-esteem could significantly predict depression. Self-efficacy, a key element of social cognitive theory, appears to be an important variable because it affects students' motivation and learning. Several factors appeared to influence students' self-efficacy (22). High levels of self-efficacy decrease a person's possibility of experiencing stress and it can help control stress situations that one may encounter (23). Self-efficacy has been found to be positively correlated with posttraumatic growth (PTG) and to moderate the relationship between post-traumatic stress disorder (PTSD) and PTG in adolescents. An improve sense of self-efficacy in adolescents could promote positive psychological transformations (24). Psychological resources, such as self-efficacy, have been found to affect individual responses to traumatic events. A strong sense of self-efficacy strengthens a person's resilience to adversity (25).

Since mental health problems in children can have consequences and costs that affect not only the individual but also the family and society, understanding the psychological problems surrounding the COVID-19 outbreak can thus pave the way for more effective prevention, education, intervention, and treatment for families, professionals, and psychologists. Furthermore, because the effects of trauma and stress caused by social crises, particularly COVID-19 disease, on individuals, families, and communities persist, they may have an impact on a variety of psychological dimensions, including lifestyle, coping strategies, quality of life, and mental health.

Therefore, concerning the prevalence of COVID-19 in Iran and the need to investigate the effects of COVID-19 pandemic on the psychosocial status of Iranian children, the present study was conducted with the following specific objectives during the outbreak of COVID-19: (a) participants' level of depression based on the CDI; (a) participants' level of self-efficacy based on the SEQ-C; (b) the association of participants' demographic characteristics with depression; and (c) the association between depression and self-efficacy.

## Methods

### Study Design and Setting

This cross-sectional study was conducted at Iranshahr University of Medical Sciences, Sistan and Baluchistan Province, Iran. A study was used to investigate the depression and self-efficacy of Iranian children and their determinant factors during the COVID-19 pandemic.

### Sampling and Sample Size

The sampling was done by creating an online questionnaire and distributing it through social media (WhatsApp, Telegram, ETA, Soroush, and I-Gap). All children with 8 to 17 years living in both urban and rural areas in the province of Sistan and Baluchistan met the inclusion criteria. The study did not include questionnaires that were incomplete. The study population included school students at the time of data collection (*n* = 20,000). The sample size was estimated to be *n* = 313 using the Cochran formula (α = 0.05, d = 0.055, Z = 1.94). Considering a 5% dropout probability, 329 students were selected.


$$\begin{array}{l} N=\frac{N{\left(Z_{a/2}\right)}^{2}p_{q}}{\left(N-1\right)D^{2}+PQ{\left(Z_{a/2}\right)}^{2}}\\ \;=\frac{20000\;\times \;{\left(1.96\right)}^{2}\;\times \;0.25}{19999\;\times \;{\left(0.055\right)}^{2}+0.25\;\times \;{\left(1.96\right)}^{2}}=313 \end{array}$$

### Measurement

Data were collected using three questionnaires, including socio-demographic form, Children's Depression Inventory (CDI) and Self-Efficacy Questionnaire for Children (SEQ-C).

### Socio-Demographic Form

(a) Participants' demographic information includes age, gender, education level, number of siblings, parents' level of education, job, and employment status, parents working in the healthcare system, and income of the family.

(b) Participants' COVID-19 disease information includes exposure to COVID-19 disease, precautions against the COVID-19 infection, concern about COVID-19 infection, illness in the family, having symptoms of the COVID-19 disease, and how information about COVID-19 disease was obtained.

### Children's Depression Inventory

The CDI Questionnaire was developed by Kovacs and Beck (26) to measure depression in children and adolescents aged 7–17 years. The CDI scale consists of 27 items with five subscales of negative mood (6 items), interpersonal problems (4 items), ineffectiveness (4 items), anhedonia (8 items) and negative self-esteem (5 items). Each question of CDI consists of three sentences to measure depressive symptoms such as crying, suicidal ideation and the ability to focus on homework. Each item is assigned a score ranging from zero to two. The participants choose one of the three sentences that express his / her feelings and thoughts during the last 2 weeks. Questions are graded on a scale of 0 to 2. A score of zero indicates that there is no sign; a score of one indicates that there is a moderate sign, and a score of two indicates that there is an obvious sign. In Mokhtarnia et al. (27) study, Cronbach's alpha for this scale was reported to be 0.78 in Iranian children. In the present study, the Cronbach's alpha for CDI was 0.76.

### Self-Efficacy Questionnaire for Children (SEQ-C)

To measure self-efficacy in children and adolescents, Muris used SEQ-C, a 23-item questionnaire, which included eight social questions, eight educational questions, seven emotional questions, and a general question. Five items (ranging from one to five) are used in each question of the SEQ-C to measure a person's level of self-efficacy. Cronbach's alpha coefficient is set to 0.86 in the original version (28). Habibi et al. investigated the psychometric properties of this scale in Iran (2014). The construct validity of this scale was confirmed by confirmatory factor analysis, and its convergent validity was confirmed by the Children Depression Inventory. In addition, Cronbach's alpha coefficients for social, educational, emotional factors and the whole scale were 0.73, 0.82, 0.76, and 0.85, respectively (29). In the present study, the Cronbach's alpha values for social, educational, emotional factors and the whole scale of CDI were 0.76, 0.89, 0.85, and 0.90, respectively.

### Data Collection

Following the acquisition of the necessary permissions, the research team and computer experts created an online questionnaire based on the content of demographic information, COVID-related items, CDI, and SEQ-C. The research team controlled and tested the efficiency and responsiveness of this online questionnaire on 30 children. Data was collected from August to October 2020 in the third wave of coronavirus outbreaks. The questionnaire was distributed in coordination with schools in school social groups in urban and rural areas. The questionnaire was reported by 8–17-year-old children themselves and was completed by parents asking questions if the children had difficulty reading the questions (Only 7 questionnaires were completed by parents). Thirty-one hundred enO and sixty-seven questionnaires were studied, with 46 incomplete questionnaires removed (effective response rate: 87.46%). Data from 321 participants was used in the final analysis after the exclusion of incomplete questionnaires.

### Data Analysis

SPSS 22 was then used to analyze the data. The data was described using descriptive statistics (frequency, percent, mean, and standard deviation). The correlation between the quantitative variables of the study was determined by the Pearson correlation coefficient. Independent *t*-test and ANOVA tests were used to determine SEQ-C based on the qualitative variables of the study. The SEQ-C determinants were identified using multivariate linear regression. The significance level of 0.05 was used.

### Ethical Considerations

This research, with a code of ethics No. IR.IRSHUMS.REC.1399.008 was approved by Iranshahr University of Medical Sciences. The objectives and methodology of the study were explained to the selected parents of a child. Consent form and questionnaires were sent to school social groups and it was mentioned that participating in the study is voluntary. After filling out the written consent form, the participants completed the first part of the online questionnaire anonymously. The written consent forms containing information on the objectives of the study, confidentiality, and exclusion from the study in the case of dissatisfaction were provided to all the participants. Participants agreed that their information would be used in research.

## Results

The mean age of children was 14.14 ± 1.91 (min = 8 to max = 17) years. The majority of the participants were middle school (62.6%) girls (75.7%) aged 13–17 years who had two siblings (26.8%) (Table 1). Other results of demographic characteristics of the participants are presented in Table 1. 8.4% of the participants were infected with the coronavirus (*n* = 27) and 57.6% of participants had relatives or friends who were infected with the coronavirus (*n* = 185). Furthermore, 38.6% of participants (*n* = 124) considered themselves to be at risk of the COVID-19 disease, and 75.1% of participants considered the effects of preventive measures in preventing the COVID-19 disease to be significant. More than 58% of participants (*n* = 187) said they always took precautions to avoid COVID-19 infection and practiced hand washing and disinfection (*n* = 187). The majority of the participants (41.1%) always advised others to take preventative measures. More than 35% of the participants (*n* = 114) were concerned about whether their families had been infected with COVID-19. In addition, 46.1% of the participants obtained the information about the COVID-19 disease from social networks (*n* = 148) (Table 2).

**Table 1:** Demographic characteristics of the participants and their associations with depression (*n* = 321).

**Variables**	**Frequency (Valid percent)**	**Depression score**		**Statistical test (*p*-value)**
		**Mean**	**SD**	
**Age**				
8–12	47 (14.6)	24.68	4.02	*t* = −1.14 (0.25)
13–17	274 (85.4)	25.39	3.96	
**Gender**				
Boy	78 (24.3)	24.62	4.99	*t* = −1.70 (0.09)
Girl	243 (75.7)	25.50	3.57	
**Level of education**				
Primary	27 (8.4)	24.37	3.79	*F* = 1.18 (0.30)
Middle school	201 (62.6)	25.51	4.11	
Secondary school	93 (29.0)	25.07	3.71	
**Sibling**				
0	7 (2.2)	21.85	5.69	*F* = 1.31 (0.25)
1	40 (12.5)	25.80	3.00	
2	86 (26.8)	25.13	4.26	
3	79 (24.6)	25.29	4.35	
4	49 (15.3)	24.95	3.98	
5	39 (12.1)	25.64	3.09	
≥6	21 (6.5)	26.23	3.44	
**Father's job**				
Employed	115 (35.8)	25.11	4.06	*F* = 1.24 (0.29)
Self-employed	126 (39.3)	25.71	3.72	
Unemployed - retired	80 (24.9)	24.88	4.20	
**Mother's job**				
Employed	83 (25.9)	25.89	3.79	*F* = 1.37 (0.25)
Self-employed	42 (13.1)	25.33	4.83	
Housewife - unemployed - retired	196 (61.1)	25.03	3.84	
**Income of family (Million Toman)^*†*^**				
<1	75 (23.4)	24.58	3.86	*F* = 3.43 (0.017)
1–3	85 (26.5)	25.30	3.44	
3–5	78 (24.3)	24.78	4.69	
>5	83 (25.9)	26.39	3.65	
**Father working in the health system**				
Yes	49 (15.3)	25.40	3.93	*t* = 0.22 (0.82)
No	272 (84.7)	25.27	3.99	
**Mother working in the health system**				
Yes	27 (8.4)	25.33	2.93	*t* = 0.05 (0.95)
No	294 (91.6)	25.28	4.06	

*SD, Standard Deviation; t, Independent t test; F, Analysis of variance*;

† *, One Dollar was 25000 Tomans*.

**Table 2:** COVID-19 related variables and their associations with depression among children (*n* = 321).

**Variables**	**Frequency (Valid percent)**	**Depression score**		**Statistical test (p-value)**
		**Mean**	**SD**	
**Infected with the coronavirus**				
Yes	27 (8.4)	24.33	7.14	*t* = −0.75 (0.45)
No	294 (91.6)	25.38	3.55	
**Relatives/ friends infected with the coronavirus**				
Yes	185 (57.6)	25.20	4.61	*t* = −0.49 (0.62)
No	136 (42.4)	25.41	2.90	
**Being at risk of coronavirus infection**				
Yes	124 (38.6)	24.65	4.87	*t* = −2.10 (0.036)
No	197 (61.4)	25.69	3.24	
**Effectiveness of precautionary measures**				
Low	15 (4.7)	22.46	6.15	*F* = 4.23 (0.015)
Medium	65 (20.2)	25.16	4.77	
Much	241 (75.1)	25.50	3.50	
**Taking precautions to prevent coronavirus infection**				
Seldom / sometimes	36 (11.2)	24.02	6.14	*F* = 2.20 (0.11)
Most of the time	98 (30.5)	25.62	3.49	
Always	187 (58.3)	25.36	3.65	
**Adherence to hand washing and disinfection**				
Seldom / sometimes	40 (12.4)	25.07	6.59	*F* = 1.21 (0.29)
Most of the time	94 (29.3)	25.82	3.02	
Always	187 (58.3)	25.06	3.64	
**Advising others to take preventative measures**				
Seldom / sometimes	87 (27.1)	25.36	4.49	*F* = 0.77 (0.43)
Most of the time	102 (31.8)	25.62	3.90	
Always	132 (41.1)	24.98	3.66	
**The most important concern about the coronavirus**				
My family getting sick	114 (35.5)	25.22	3.63	*F* = 0.02 (0.97)
Death	89 (27.7)	25.33	4.16	
Others	118 (36.8)	25.32	4.18	
**How information about the coronavirus disease was obtained**				
Internet	78 (24.3)	24.12	4.90	*F* = 3.38 (0.018)
Medical staff	49 (15.3)	25.38	3.25	
Social networks	148 (46.1)	25.87	3.40	
Others	46 (14.3)	25.28	4.30	

*SD, Standard Deviation; t, Independent t test; F, Analysis of variance*.

The mean score of depression was 25.29 ± 3.97, which was higher than the midpoint of the questionnaire (score = 13.5). Among the CDI subscales, the anhedonia had the highest score, while interpersonal problems had the lowest score. The mean score of self-efficacy was 79.84 ± 14.18, which was greater than the midpoint of the questionnaire (score = 57.5). Among self-efficacy subscales, the academic subscale had the highest score and emotional subscale had the lowest score (Table 3).

**Table 3:** Description of the depression and self-efficacy and their dimensions' scores among children (*n* = 321).

**Variable**		**Median**	**Mean**	**SD**	**Minimum**	**Maximum**
Depression	Negative mood	7.00	6.43	1.72	1	10
	Interpersonal problems	2.00	2.62	1.17	0	7
	Ineffectiveness	3.00	3.42	1.54	0	7
	Anhedonia	7.00	6.60	1.85	0	15
	Negative self-esteem	7.00	6.19	1.66	1	9
	Total	26.00	25.29	3.97	4	41
Self-efficacy	Social	27.00	27.25	5.46	11	40
	Academic	31.00	30.38	6.07	10	40
	Emotional	22.00	22.20	5.88	7	35
	Total	81.00	79.84	14.18	35	114

*SD, Standard Deviation*.

No significant correlation was observed between depression and self-efficacy of children (*P* = 0.23; *r* = 0.06). However, in subscale of CDI, negative mood (*r* = 0.31), ineffectiveness (*r* = 0.-41) and negative self-esteem (*r* = 0.33) had a significant correlation with self-efficacy (*p* < 0.001). None of self-efficacy subscales had a significant correlation with depression (Table 4).

**Table 4:** Correlation between depression and self-efficacy and their dimensions' scores among children (*n* = 321).

**Variable**		**Self-efficacy (Pearson's correlation coefficient)**			
		**Social**	**Academic**	**Emotional**	**Total**
Depression	Negative mood	0.27^*^	0.21^*^	0.28^*^	0.31^*^
	Interpersonal problems	0.02	0.02	0.01	0.02
	Ineffectiveness	−0.29^*^	−0.36^*^	−0.34^*^	−0.41^*^
	Anhedonia	−0.12^***^	−0.03	−0.15^**^	−0.12^***^
	Negative self-esteem	0.26^*^	0.24^*^	0.31^*^	0.33^*^
	Total	0.06	0.04	0.05	0.06

* *p < 0.001*;

** *p < 0.01*,

*** *p < 0.05*.

The bivariate analysis showed a significant correlation between depression, income of family (*p* = 0.017), being at risk of coronavirus infection (*p* = 0.036), effectiveness of precautionary measures (*p* = 0.015) and how information about the coronavirus disease was obtained (*p* = 0.018) (Tables 1, 2).

## Discussion

The purpose of this study was to look into depression and self-efficacy in Iranian children during the COVID-19 outbreak. The results of the current study revealed that the mean score of depression was higher than the midpoint of the questionnaire in children, indicating that children experienced depression during the COVID-19 epidemic. Anhedonia had the highest score in children among the depression subscales, while interpersonal problems had the lowest score. Bignardi et al. (30) emphasized that during the COVID-19 outbreak, symptoms of childhood depression were much higher during quarantine in the UK than before quarantine. These results support the current study's results. Kumar Panda et al. (31) identified anxiety, depression, irritability, impatience, inattention, and fear of COVID-19 as psychological problems in children during the COVID-19 outbreak. They also discovered that behavioral symptoms in children who already had behavioral problems, such as autism and attention-deficit/hyperactivity disorder were more likely to get worse (31). De Miranda et al. (32) discovered that children responded to stress in different ways depending on their developmental stage. As a result, high levels of anxiety, depression, and posttraumatic symptoms were found in children during the outbreak of COVID-19 (32). These results are consistent with the results of the current study. McKune et al. (33) found that the COVID-19 outbreak, followed by children quarantine, caused symptoms such as anxiety, depression, and obsessive-compulsive disorder. They admitted that these factors were more prevalent in younger children, girls, and low-income families. They also stated that school quarantine during the COVID-19 outbreak might have negative effects on children's mental health, so school principals should identify those at risk as soon as possible and take steps to reduce these risks (33). Yue et al. (34) considered anxiety, depression, and posttraumatic stress disorder as psychological problems for children and their parents during the COVID-19 outbreak. In this study, children living in areas with a lower prevalence of COVID-19 had fewer psychological consequences (34). Tang et al. (35) discovered three common psychological symptoms in children during the COVID-19 outbreak: anxiety, depression, and stress. They found that children who discussed COVID-19 with their parents experienced less anxiety, depression, and stress (35). Mangolian Shahrbabaki et al. (36) reported moderate-to-severe anxiety in female children who participated in their study. According to the findings of this study, 7–11-year-old girls suffered from fear and anxiety about COVID-19 because they misunderstood the news on social media and networks. On the other hand, parents could reduce their children's fear and anxiety by using simple words and explaining the disease to them (36).

According to the results of this study, the mean self-efficacy score was higher than the questionnaire's midpoint, indicating that the level of self-efficacy in the children who participated in this study was relatively acceptable. According to the articles in this field, no study examined self-efficacy in children during the coronavirus outbreak, so the studies that were most consistent with the current study were used to discuss and explain the results. As a result, Kermansaravi et al. (37) concluded that adolescents with type 1 diabetes had moderate self-efficacy. According to Behnam Vashani et al. (38), the self-efficacy of children with thalassemia major was moderate. Sheibani et al. (39), on the other hand, stated that the rate of self-efficacy in adolescents with thalassemia major was low. The results of these studies may contradict to the results of the current study. Differences in results may be due to differences in target group age, target population, and data collection tools.

Self-efficacy beliefs determine how people feel, think, motivate themselves and behave (37). Self-efficacy, a key element of social cognitive theory, appears to be an important variable because it affects students' motivation and learning (38). A strong sense of efficacy enhances human accomplishment and personal wellbeing in many ways. Such an efficacious outlook produces personal accomplishments, reduces stress and lowers vulnerability to depression (39). Early stressful experiences may be related to the development of psychopathologies such as depression and social anxiety in adulthood (40). Various aspects of mental health are influenced by the sense of self-efficacy appraisal. As a result, low self-efficacy usually exacerbates some problems, such as emotional and social issues associated with mental health (41).

Depression, alcohol abuse, and suicidality each continue to threaten adolescent populations throughout the world. The comorbidity between these diseases has been found to be up to 73%, with consistent positive correlations between adolescent drinking, depression, and suicidality (40). Common risk factors for adolescent suicidality include depression and conduct problems (42). It is supposed that increasing the sense of self-efficacy helps manage such unpleasant emotions better and so decrease the probable harmful outcomes. Some research in this area has addressed the role of self-efficacy in the early-onset of depression. Children's perceived social and academic inefficacy contributed to concurrent and subsequent depression both directly and indirectly through their impact on academic achievement, prosociality, and problem behaviors (39). According to a study by Sawatzky et al. (43), identifying students with limited stress management self-efficacy and providing them with appropriate supportive services may help them to manage stress and prevent depression (44). By examining the determinants of depression and self-efficacy, a deeper understanding of children's conditions in crises such as the coronavirus can be achieved.

According to the results of this study, there is no significant correlation between children's depression and self-efficacy. In the CDI subscale, however, negative mood, inefficiency, and negative self-esteem were all significantly related to self-efficacy. However, none of the self-efficacy subscales had a significant correlation with depression. Hong et al. (21) demonstrated that the emotional climate of the classroom could have a significant impact on social self-efficacy and that social self-efficacy could predict self-esteem and depression. Marle et al. (41) also discussed the role of self-efficacy as a protective factor against depression caused by COVID-19. Wen et al. (45) showed that increasing positive self-esteem, such as hope and self-efficacy, reduced students' stress during COVID-19. The findings of the previous studies contradict the findings of the current study. Differences in sample size, study population, research setting, and data collection tools may all be reasons for inconsistency. The type of psychological disorder in Wen et al.'s study was also different from that in the current study. According to Alemany-Arrebola et al. (42), a stressful situation (such as pandemic and quarantine) combined with a critical event (illness / death of a relative / friend due to COVID-19) increased students' anxiety and thus affected their academic self-efficacy. This study is also contradictory to the current study. It should be noted that in this study, students' anxiety was measured using self-efficacy, which could explain why the results of the two studies differed. Liu et al. (44) discovered a negative correlation between anxiety, depression, and self-efficacy in children with malignant tumors, and that increasing self-efficacy could reduce anxiety and depression. The findings of this study contradict the findings of the current study. Children with malignant tumors were examined in this study, which could explain why the results of the two studies differed. Furthermore, there were differences in sample size and data collection tools between the two studies.

Negative mood, interpersonal problems, ineffectiveness, lack of pleasure, and low self-esteem were all predictors of depression in the current study. Hong et al. (21) emphasized the current study's findings, demonstrating that self-esteem could significantly predict depression. However, due to a lack of additional research in this area, researchers must pay closer attention to the predictors of depression in children, particularly in crises such as COVID-19 disease.

The findings of this study revealed that the prevalence of COVID-19 and its associated factors, such as disturbances in daily routines and way of life, have produced concerns and problems among vulnerable populations, such as children. Consequently, the position of children and the trend of the COVID-19 outbreak in the region must be taken into account while managing vulnerable populations such as children. In the present study, bad mood, inefficiency, and low self-esteem were connected with self-efficacy in children, which should be taken into account in future research. There is a need for effective approaches to lessen childhood depression and prevent the long-term effects of COVID-19 outbreaks and worldwide crises. This study's findings can assist in identifying the levels of depression in children with COVID-19 as a worldwide crisis, as well as possible elements that contribute to the development of children's self-efficacy in professional guidance and crisis preparation involving COVID-19. Concerning future directions, we point to the existing position of children in crisis, which must be taken into account in order to combat the psychological consequences of epidemics and crises. Future research will focus on aspects associated with children's self-efficacy in times of global catastrophe. In the future, understanding and advancing research on how to boost children's self-efficacy during times of global crisis or pandemic may be an essential field of study.

## Limitations

The cross-sectional research design limits our understanding of the overall risk factors for depression and prevents us from determining the causal relationships between the variables studied. Longitudinal studies are required. As a result, longitudinal or interventional research should be conducted in the future. Second, self-report questionnaires were used, implying that future studies will require evaluations that are more specialized. Third, because of respondents' attitudes toward themselves, the use of self-report tools may result in biased answers to questions, which should be interpreted with caution. Our sampling was performed in the third wave of coronavirus outbreaks and was not related to all outbreaks of the disease, so the results may have been affected. Finally, because data were collected online without an independent assessment of respondents' health status, results should be generalized with caution.

One of the strengths of the present study is the attempt to better understand the factors affecting depression in children in the coronavirus crisis. Due to the special conditions and quarantine of children and the existence of various restrictions that make it difficult to have direct contact with children, the present study presents the challenges regarding the self-efficacy of children.

## Conclusion

The current study found that the rate of depression and self-efficacy in children during the COVID-19 outbreak was higher than average, but self-efficacy did not play a significant role in predicting depression. Given that children are one of the most vulnerable groups in society, psychological trauma and problems can have a negative impact on them and society in the future. Furthermore, because pandemics such as COVID-19 will have a long-term impact on communities, appropriate and comprehensive planning is required to reduce psychological problems, particularly depression in children. Further studies in this area are needed since the children in this study had high self-efficacy and no correlation between self-efficacy and depression was found.

## Data Availability Statement

The raw data supporting the conclusions of this article will be made available by the authors, without undue reservation.

## Ethics Statement

The studies involving human participants were reviewed and approved by Iranshahr University of Medical Sciences No. IR.IRSHUMS.REC.1399.008. Written informed consent to participate in this study was provided by the participants' legal guardian/next of kin.

## Author Contributions

MZ, AD, and MD contributed to conception and design of the study. AD, AS, MA, and MK contributed to data collection. MZ and MD performed statistical analyses. MZ, MD, and FS wrote the first draft of the manuscript. All authors contributed to manuscript revision, read, and approved the submitted version.

## Conflict of Interest

The authors declare that the research was conducted in the absence of any commercial or financial relationships that could be construed as a potential conflict of interest.

## Publisher's Note

All claims expressed in this article are solely those of the authors and do not necessarily represent those of their affiliated organizations, or those of the publisher, the editors and the reviewers. Any product that may be evaluated in this article, or claim that may be made by its manufacturer, is not guaranteed or endorsed by the publisher.

## References

[1] Liu Y Sun W Chen L Wang Y Zhang L Yu L. Clinical characteristics and progression of 2019 novel coronavirus-infected patients concurrent acute respiratory distress syndrome. MedRxiv. (2020). 10.1101/2020.02.17.20024166

[2] Kunling S Yonghong Y Tianyou W Dongchi Z Yi J Runming J. Diagnosis, treatment, and prevention of 2019 novel coronavirus infection in children: experts' consensus statement. World J Pediatr. (2020) 16:223–31. 10.1007/s12519-020-00343-7 32034659 PMC7090771

[3] Zakeri MA Rafsanjanipoor SMH Sedri N Kahnooji M Rafsanjani MS Zakeri M. Psychosocial status during the prevalence of COVID-19 disease: the comparison between healthcare workers and general population. Curr Psychol. (2021) 40:6324–32. 10.1007/s12144-021-01582-1 33746463 PMC7963465

[4] Zakeri MA SM HR Kahnooji M Dehghan M. Generalized anxiety disorder during the COVID-19 outbreak in Iran: the role of social dysfunction. J Nerv Ment Dis. (2021) 209:491–6. 10.1097/NMD.0000000000001320 33600121

[5] Hossini Rafsanjanipoor SM Zakeri MA Dehghan M Kahnooji M Sanji Rafsanjani M Ahmadinia H. Iranian psychosocial status and its determinant factors during the prevalence of COVID-19 disease. Psychol Health Med. (2021) 27:30–41. 10.1080/13548506.2021.1874438 33486996

[6] Zakeri MA Maazallahi M Ehsani V Dehghan M. Iranian psychosocial status during and after COVID-19 outbreak mandatory quarantine: a cross-sectional study. J Commun Psychol. (2021) 49:2506–16. 10.1002/jcop.22647 34237163

[7] Shirzadi P Shirazi NA Lashkami ZA. Relationship between corona anxiety in mothers and parent-child interaction and children's aggression during quarantine days. J Fam Res. (2020) 16:139–54. Available online at: https://jfr.sbu.ac.ir/article_97811.html

[8] choobdari a Nikkhoo F Fooladi F. Psychological consequences of new coronavirus (Covid 19) in children: a systematic review. Quart Edu Psychol. (2020) 16:51–63. 10.22054/JEP.2020.53306.3043

[9] Fegert JM Vitiello B Plener PL Clemens V. Challenges and burden of the Coronavirus 2019 (COVID-19) pandemic for child and adolescent mental health: a narrative review to highlight clinical andresearch needs in the acute phase and the long return to normality. Child Adolesc Psychiatry Ment Health. (2020) 14:1–11. 10.1186/s13034-020-00329-3 32419840 PMC7216870

[10] Roccella M. Children and coronavirus infection (Covid-19): what to tell children to avoid post-traumatic stress disorder (PTSD). Open Pediatr Med Journal. (2020) 10:14–9. 10.2174/1874309902010010001

[11] Ghosh R Dubey MJ Chatterjee S Dubey S. Impact of COVID-19 on children: special focus on psychosocial aspect. Minerva Pediatr. (2020) 72:226–35. 10.23736/S0026-4946.20.05887-9 32613821

[12] Zakeri MA Dehghan M Heidari FG Pakdaman H Mehdizadeh M Ganjeh H. Mental health outcomes among health-care workers during the COVID-19 outbreak in Iran. Ment Health Rev J. (2021) 26:152–60. 10.1108/MHRJ-10-2020-0075

[13] Wang G Zhang Y Zhao J Zhang J Jiang F. Mitigate the effects of home confinement on children during the COVID-19 outbreak. Lancet. (2020) 395:945–7. 10.1016/S0140-6736(20)30547-X 32145186 PMC7124694

[14] Ouweneel E Schaufeli WB Le Blanc PM. Believe, and you will achieve: changes over time in self-efficacy, engagement, and performance. Appl Psychol Health Well-Being. (2103) 5:225–47. 10.1111/aphw.12008 23616308

[15] Morelli M Cattelino E Baiocco R Trumello C Babore A Candelori C. Parents and children during the COVID-19 lockdown: the influence of parenting distress and parenting self-efficacy on children's emotional well-being. Front Psychol. (2020) 11:584645. 10.3389/fpsyg.2020.584645 33123063 PMC7574609

[16] Zhou C Yue XD Zhang X Shangguan F Zhang XY. Self-efficacy and mental health problems during COVID-19 pandemic: a multiple mediation model based on the health belief model. Pers Individ Dif. (2021) 179:110893. 10.1016/j.paid.2021.110893 PMC9756413 36540084

[17] Okan N. Investigating the moderator effect of fear of COVID-19 in the relation between communication anxiety and self-efficacy. Educ Proces Int J. (2021) 10:62–77. 10.22521/edupij.2021.103.5

[18] Schwarzer R. Self-Efficacy: Thought Control of Action. 1st ed, New York: Taylor & Francis (2014). 10.4324/9781315800820

[19] Bandura A. Regulative Function of Perceived Self-Efficacy. Personnel Selection and Classification. 1st ed, New York: Psychology Press (2013). p. 279–90.

[20] Chang C-S Liu EZ-F Sung H-Y Lin C-H Chen N-S Cheng S-S. Effects of online college student's Internet self-efficacy on learning motivation and performance. Innov Educ Teach Int. (2014) 51:366–77. 10.1080/14703297.2013.771429

[21] Hong F Chiu S Huang D. Correlations among classroom emotional climate, social self-efficacy, and psychological health of university students in Taiwan. Educ Urban Soc. (2021) 53:446–68. 10.1177/0013124520931458

[22] Van Dinther M Dochy F Segers M. Factors affecting students' self-efficacy in higher education. Educ Res Rev. (2011) 6:95–108. 10.1016/j.edurev.2010.10.003

[23] Shahrour G Dardas LA. Acute stress disorder, coping self-efficacy and subsequent psychological distress among nurses amid COVID-19. J Nurs Manag. (2020) 28:1686–95. 10.1111/jonm.13124 32767827 PMC7436502

[24] Jian Y Hu T Zong Y Tang W. Relationship between post-traumatic disorder and posttraumatic growth in COVID-19 home-confined adolescents: the moderating role of self-efficacy. Curr Psychol. (2022) 1–10. 10.1007/s12144-021-02515-8 35018083 PMC8736319

[25] Benight CC Bandura A. Social cognitive theory of posttraumatic recovery: the role of perceived self-efficacy. Behav Res Ther. (2004) 42:1129–48. 10.1016/j.brat.2003.08.008 15350854

[26] Kovacs M Beck AT. An Empirical-Clinical Approach Toward a Definition of Childhood Depression. Depression in Childhood: Diagnosis, Treatment, and Conceptual Models. (1977). p. 1–25.

[27] Mokhtarnia I Habibi M Kholghi H Mohammadi E Kalantari F. The Study of psychometric properties of the self-rating depression scale for children and adolescents. Rooyesh-e-Ravanshenasi J. (2018) 7:1–22. Available online at: http://frooyesh.ir/article-1-296-fa.html

[28] Muris P. A brief questionnaire for measuring self-efficacy in youths. J Psychopathol Behav Assess. (2001) 23:145–9. 10.1023/A:1010961119608

[29] Habibi M Tahmasian K Ferrer-Wreder L. Self-efficacy in persian adolescents: psychometric properties of a Persian version of the self-efficacy questionnaire for children (SEQ-C). Int Perspect Psychol. (2014) 3:93–105. 10.1037/a0036059

[30] Bignardi G Dalmaijer E Anwyl-Irvine A Smith TA Siugzdaite R Uh S. Longitudinal increases in childhood depression symptoms during the COVID-19 lockdown. Arch Dis Child. (2020) 106:791–7. 10.1136/archdischild-2020-320372 33298552 PMC7733224

[31] Kumar Panda P Gupta J Chowdhury S Kumar R Meena AK Madaan P. Psychological and behavioral impact of lockdown and quarantine measures for COVID-19 pandemic on children, adolescents and caregivers: a systematic review and meta-analysis. J Trop Pediatr. (2020) 67:1–13. 10.1093/tropej/fmaa122 33367907 PMC7798512

[32] de Miranda DM da Silva Athanasio B de Sena Oliveira AC Silva ACS. How is COVID-19 pandemic impacting mental health of children and adolescents? Int J Disaster Risk Reduct. (2020) 51:101845. 10.1016/j.ijdrr.2020.101845 32929399 PMC7481176

[33] McKune S Acosta D Diaz N Brittain K Joyce- Beaulieu D Maurelli AT. Psychosocial health of school-aged children during the initial COVID-19 safer-at-home school mandates in Florida: a cross sectional study. BMC Public Health. (2021) 21:603. 10.1186/s12889-021-10540-2 33781220 PMC8006116

[34] Yue J Zang X Le Y An Y. Anxiety, depression and PTSD among children and their parent during 2019 novel coronavirus disease (COVID-19) outbreak in China. Curr Psychol. (2020) 1–8. 10.1007/s12144-020-01191-4 33223783 PMC7666617

[35] Tang S Xiang M Cheung T Xiang Y. Mental health and its correlates among children and adolescents during COVID-19 school closure: the importance of parent-child discussion. J Affect Disord. (2021) 279:353–60. 10.1016/j.jad.2020.10.016 33099049 PMC7550131

[36] Mangolian Shahrbabaki P Dehghan M Maazallahi M Asadi N. Fear and anxiety in girls aged 7 to 11 years old and related factors during the coronavirus pandemic. Clin Child Psychol Psychiatry. (2021) 27:259–68. 10.1177/13591045211013873 33951970 PMC8829152

[37] Kermansaravi F Navidian A Sargazishad T Ebrahimi Tabas E. Evaluation of self-efficacy and some related factors in adolescents with type I diabetes referred to diabetes clinic of Hazrat Ali Asghar Zahedi 2016. J Diabetes Nurs. (2017) 5:187–98. Available online at: http://jdn.zbmu.ac.ir/article-1-235-en.html

[38] Behnam Vashani H Hekmati pour N Vaghee S Asghari Nekah S. Survay of Social, Emotional and academic self efficacy in 7-12 aged children with major thalassemia in mashhad (2013). Iran Nurs Sci Assoc. (2015) 2:49–57. Available online at: http://jpen.ir/article-1-133-en.html

[39] Sheibani B Parvizi S Haghaani H Borimnejad L. The self-efficacy of adolescents with major thalassemia and its influencing factors in Bandar Abbas. Iran Nurs Sci Assoc. (2015) 1:26–33. Available online at: http://jpen.ir/article-1-43-en.html

[40] Ganz D Sher L. Suicidal behavior in adolescents with comorbid depression and alcohol abuse. Minerva Pediatr. (2009) 61:333–47. Available online at: https://pubmed.ncbi.nlm.nih.gov/19461576/19461576

[41] Marle C Parmentier F CVinchon F Storme M Borteyrou X Lubart T. Evolution and impact of self-efficacy during French COVID-19 confinement: a longitudinal study. J Gen Psychol. (2021) 148:360–81. 10.1080/00221309.2021.1904815 33825670

[42] Alemany-Arrebola I Rojas-Ruiz G Granda-Vera J Mingorance-Estrada ÁC. Influence of COVID-19 on the perception of academic self-efficacy, state anxiety, and trait anxiety in college students. Front Psychol. (2020) 11:570017. 10.3389/fpsyg.2020.570017 33154727 PMC7586314

[43] Sawatzky RG Ratner PA Richardson CG Washburn C Sudmant W Mirwaldt P. Stress and depression in students: the mediating role of stress management self-efficacy. Nursing Res. (2012) 61:13–21. 10.1097/NNR.0b013e31823b1440 22166906

[44] Liu Q Mo L Huang X Yu L Liu Y. Path analysis of the effects of social support, self-efficacy, and coping style on psychological stress in children with malignant tumor during treatment. Medicine. (2020) 99:e22888. 10.1097/MD.0000000000022888 33120834 PMC7581179

[45] Wen F Zhu J Ye H Li L-Y Ma Z Wen X-X. Associations between insecurity and stress among Chinese university students: the mediating effects of hope and self-efficacy. J Affect Disord. (2021) 15:447–53. 10.1016/j.jad.2020.12.047 33360366

